# Th2 Cytokine Levels Distort the Association of IL-10 and IFN-*γ* with Allergic Phenotypes

**DOI:** 10.5402/2011/405813

**Published:** 2011-12-28

**Authors:** Guicheng Zhang, Catherine M. Hayden, Jack Goldblatt, Patrick Holt, Peter N. Le Souëf

**Affiliations:** ^1^School of Paediatrics and Child Health, Faculty of Medicine, Dentistry and Health Sciences, The University of Western Australia, GPO Box D184, Perth, WA 6840, Australia; ^2^Telethon Institute for Child Health Research and UWA Centre for Child Health Research, Faculty of Medicine, Dentistry and Health Sciences, The University of Western Australia, GPO Box D184, Perth, WA 6840, Australia

## Abstract

The expression of allergic phenotypes involves complex inter-relationships among several Th2 and Th1 cytokines as well as the regulator cytokine interleukin (IL)-10. These direct or indirect interrelationships may distort the true associations of cytokine responses with these phenotypes. In this study, we aimed to clarify the effects of the regulatory cytokine IL-10 and Th1 cytokine interferon-gamma (IFN-*γ*) on allergic phenotypes after adjusting for the correlations with Th2 cytokines. After adjusting for Th2 cytokines, IL-10 and IFN-*γ* were protective against atopy. Adjusted levels of IL-10 and IFN-*γ* stimulated with house-dust mite (HDM) were significantly lower in atopics than non-atopics, for IL-10 adjusting for IL-5 (*P* = 0.002), IL-13 (*P* = 0.012), IL-9 (*P* = 0.016), and IL-4 (*P* = 0.043), and for IFN-*γ* adjusting for IL-5 (*P* = 0.005), IL-13 (*P* = 0.005), and IL-9 (*P* = 0.037). IL-10 and IFN-*γ* levels stimulated with phytohaemagglutinin (PHA) and staphylococcal enterotoxin B (SEB) exhibited a similar pattern. The adjusted levels of IL-10 and IFN-*γ* stimulated with HDM, PHA or SEB were all significantly negatively correlated with total serum IgE, except for IFN-*γ* stimulated with SEB. Levels of Th2 cytokines distort the associations of IL-10 and IFN-*γ* with allergic phenotypes. Removing the covariance with Th2 cytokines, both IL-10 and IFN-*γ* were protective against atopy.

## 1. Introduction 

Abnormalities in T-helper (Th)2/Th1 balance are considered to be involved in the pathogenesis of several chronic diseases including atopy and asthma. There are at least two types of T-helper cells that regulate the Th2/Th1 balance through their corresponding cytokines, which have reciprocal functions to balance the immune system and protect organisms from infection. The Th1 cytokines interferon-gamma (IFN-*γ*) and interleukin (IL)-2 activate macrophages and cytotoxic Tcells to kill intracellular organisms, while IL-5, IL-4, and other Th2 cytokines help B cells to secrete protective antibodies [1–3]. IL-10, which is produced in recently differentiated primary Th1 and Th2 cells, regulatory T cells, B cells, monocytes, macrophages, and dendritic cells, is a pleiotropic cytokine and is considered a major regulator of the homeostasis of the immune system [4, 5]. Multifaceted inflammatory processes, orchestrated and regulated by a complex network of mutually interacting cytokines, determine the prognosis of chronic disorders, such as atopy and asthma [6, 7].

Experiments on animals clearly established the concept that Th1/Th2 balance is involved in the etiology of atopy and asthma [2, 7, 8]. Th2 and Th1 cytokines, representative of the two polarized immune processes, have been extensively investigated with regard to the pathogenesis of asthma and allergy. However, the mechanisms underlying the establishment and maintenance of this Th1/Th2 balance are still poorly understood in humans. Consistent with the Th1/Th2 balance concept, allergic individuals would be expected to have enhanced Th2 immunity with a depressed Th1 response [9] and many studies have reported that atopy is associated with a Th2-dominated response, with increased IL-5, IL-13, IL-9, and IL-4 cytokine responses [10, 11]. However, the levels of the Th1 cytokine IFN-*γ* and regulator IL-10 were not found to be significantly different between atopics and non-atopics [11]. As the immune response involves complex direct/indirect interrelationships among cytokines, these interactions may distort the true associations of cytokine responses with allergic phenotypes. The present study aimed to clarify the effects of the regulatory cytokine IL-10 and Th1 cytokine IFN-*γ* on allergic phenotypes after adjusting for covariance with Th2 cytokines. We expected that atopic phenotypes would be associated with a decreased level of a Th1 cytokine such as IFN-*γ*, based on the established knowledge on Th1/Th2 balance with atopic phenotypes, however, this was not found in our previous analyses [11]. In addition, we recently found that the ratios of IL-10 and IL-5 capacities in cord blood were protective against acute respiratory infections during infancy and early childhood in a high-risk cohort. As IL-10 is a multifactorial cytokine regulating the Th1/Th2 balance, we also expected that IL-10 would be significantly associated with atopic phenotypes, possibly with a contrasting effect on expression of atopic phenotypes with Th2 cytokines. With a simple correlation for those cytokine data, we found there were “TRUE” correlations between IL-10 and IFN-*γ* and Th2 cytokines, (Tables 1 and 2). We assume that these correlations may confound finding any associations of IL-10 and IFN-*γ* with atopic phenotypes. Therefore, our present study aimed to examine our hypotheses based on the previously ascertained cytokine data by accounting for the complex inter-relationships of IL-10 and IFN-*γ* with several important Th2 cytokines.

## 2. Method

### 2.1. Population

The Perth Infant Asthma Followup (PIAF) longitudinal birth cohort has been previously described in several publications [12–14]. This study included 130 unselected probands for whom cytokine measurements were available from the 11-year review of the cohort. Phenotypes of atopy (defined by skin prick test (SPT)), total IgE, asthma, wheezing, and bronchial hyperresponsiveness (BHR) in the population have been described in previous publications [12–15]. The present study was approved by the institutional ethics committee of Princess Margaret Hospital, Perth.

### 2.2. Cytokines

As described previously [11, 16], peripheral blood mononuclear cells (PBMCs) responses were determined on cryobanked samples. The PBMC were cultured for 48 h in AIM-V medium with 4∗10^−5^ M 2-ME alone or with 10 *μ*g/mL house-dust mite extract or purified Der P1 dust mite allergen at 30 *μ*g/mL, or 1 *μ*g/mL phytohaemagglutinin A (PHA), or staphylococcal enterotoxin B (SEB). Cell pellets were resuspended in RNALater (Ambion, Austin) and stored at –20°C. Levels of cytokines IL-5, IL-13, IL-10, and IFN-*γ* were determined by means of ELISA or time-resolved fluorescence in cell culture supernatants. For HDM culture, IL-9 and IL-4 levels were also determined. 

### 2.3. Statistical Analysis

Levels of cytokine and total serum IgE were natural log-transformed prior to further parametric analyses. Pearsons'correlations were employed to investigate the relationships between cytokines. As shown in (Tables 1 and 2), there were significant correlations between IL-10 and IFN-*γ* with Th2 cytokines indicating that there was a direct/indirect correlation between them. Thus, a proportion of IL-10 and IFN-*γ* levels may be explained by the direct/indirect relationship with Th2 cytokines. The positive co-relationships (covariance) of IL-10, and IFN-*γ* with Th2 cytokines: IL-5, IL-13, IL-9, and IL-4 may tend to result in atopic subjects with relatively higher levels of IL-10 and IFN-*γ*. This may distort the true picture of association of the two cytokines with atopic phenotypes. To adjust for the covariance, we employed two statistical methods. In method one, adjusted levels of IL-10 and IFN-*γ* were computed by adding residuals to the mean of raw log values of the cytokines for individual subjects [17]. The residual was estimated in a linear regression model with either log value of IL-10 or log value of IFN-*γ* as dependent variable and one Th2 cytokine as independent variable (adjusted for). The adjusted IL-10 and IFN-*γ* values were further used for analyses to investigate the association with allergic phenotypes. In method two, we fitted either IL-10 (log value) or IFN-*γ* (log value) and one Th2 cytokine (adjusted for) in a logistic regression model to estimate the adjusted odds ratios (OR) and their 95% confidence intervals (95% CI) for binary atopic phenotypes. Independent sample *t*-test was utilized to explore the difference in the adjusted IL-10 and IFN-*γ* levels for the binary phenotypic variables. Pearson correlations were also conducted between adjusted IL-10 and IFN-*γ* and total serum IgE. All the analyses were performed using the software SPSS Window 16.

## 3. Results

This study included 130 children. The mean age of the children was 11.2 yrs (95% CI: 11.0–11.3 yrs). There were 55 girls (42.3%) in the population. The correlation coefficients between natural log values of cytokines IL-5, IL-13, IL-9, IL-4, IL-10, and IFN-*γ* are shown in (Tables 1 and 2). There were significant positive correlations between cytokines; unexpectedly, such correlations were also significant between Th2 cytokines, IL-10, and IFN-*γ*.

### 3.1. Adjusted Means of IL-10 and IFN-*γ* with Allergic Phenotypes

Regarding the raw means of IL-10 and IFN-*γ* stimulated with HDM, no significant difference was found between individuals with and without atopy. However, after adjusting for individual Th2 cytokines, the differences in levels of IL-10 and IFN-*γ* between atopic and non-atopic children were significant for IL-10 adjusting for IL-5 (*P* = 0.002), IL-13 (*P* = 0.012), IL-9 (*P* = 0.016), and IL-4 (*P* = 0.043), respectively, and for IFN-*γ* adjusting for IL-5 (*P* = 0.005), IL-13 (*P* = 0.005), and IL-9 (*P* = 0.037), respectively (Table 3). Atopic subjects appeared to have low levels of IL-10 and IFN-*γ* compared with non-atopics.

Consistent with the findings on IL-10 and IFN-*γ* stimulated with HDM, IL-10 and IFN-*γ* stimulated with PHA and SEB exhibited a similar pattern (Table 4). Except for IFN-*γ* (SEB), the difference IL-10 and IFN-*γ* was more apparent after adjustment IL-5 and IL-13.

We correlated the IL-10 and IFN-*γ* levels with the levels of total serum IgE. The adjusted IL-10 and IFN-*γ* were significantly negatively correlated with total IgE, but not significantly raw levels of the two cytokines. The pattern was similar for allergen-specific cytokine response as well as polyclonal stimulator response (Table 5).

The association of adjusted IL-10 and IFN-*γ* with asthma, wheezing, and bronchial hyperresponsiveness was also investigated in this population. IL-10 and IFN-*γ* were not significantly associated with these phenotypes in the whole population either before or after the adjustment for Th2 cytokines. 

### 3.2. Odds Ratios of IL-10 and IFN-*γ* for Atopy

As described in the statistical analysis section, we further investigated the association of IL-10 and IFN-*γ* with atopy in the logistic regression analysis using the two adjusted methods. Before the adjustment, IL-10 and IFN-*γ* were not significantly associated with atopy except for raw levels of PHA IL-10 and IFN-*γ*. However, after the adjustment, both IL-10 and IFN-*γ* were clearly protective against atopy. The two adjusted methods gave very similar results (Table 6). 

## 4. Discussion

**Figure 1:**
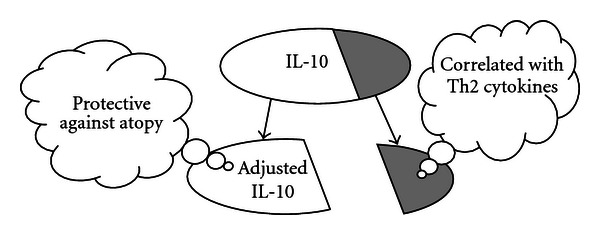
Illustrations of the major finding in the present study for IL-10.

The present study is the first to investigate the relationships of Th1 and regulatory cytokines with atopic phenotypes after adjusting for the correlative effects of Th2 cytokines in a community population. In this study we found that IL-10, major findings shown in (Figure 1), and IFN-*γ* were protective against atopy after accounting for their correlations with Th2 cytokines. After taking out the shaded area of IL-10 (adjusting for Th2 cytokines), the remaining IL-10 (leftover unshaded area) is protective against atopy. These findings provide important insights into better understanding the function of the Th1 cytokine IFN-*γ* and regulatory cytokine IL-10. 

Th2/Th1 balance was postulated after the discovery of the mutual inhibitory effects of Th1 and Th2 cells in mice, thus, a negative correlation would be expected between Th1 and Th2 cytokines [2, 8]. However, as we have shown, there was a strong positive correlation between IL-10 and IFN-*γ* and Th2 cytokines. Consistent with our findings, a recent publication reported all cytokines including Th1/Th2 groups showed marked positive intraindividual correlations [18]. There are several possible explanations for these positive correlations: childhood immunity is primed by environmental factors in specific domiciliary environments (nutrients, environmental allergen exposure, and infections). This process may, in part, contribute to such positive correlations between cytokines. Furthermore, in the process of pathogenesis of atopic conditions, polarized Th2 cytokine increases may result in increased Th1 cytokines and regulatory cytokines through a self-balance/correction mechanism. Whatever the reasons for these correlations, they may distort the associations between Th1, regulatory cytokines, and allergic phenotypes. Indeed, in the present study, after adjusting for the Th2 cytokines IL-5, IL-13, or IL-9 we found that both IL-10 and IFN-*γ* levels were significantly and consistently lower in atopic subjects. It is not unexpected that Th1 cytokines and IL-10 have a protective effect on allergy-related phenotypes. However, if cytokine data in a population report that IL-10 and IFN-*γ* are at risk for allergy and asthma, the confounding effects of Th2 cytokines should be accounted and the corresponding findings should be interpreted with caution. 

As we found that adjusting for Th2 cytokines clarified the effects of IL-10 and IFN-*γ* on atopy, we further examined the consequence of mutually adjusting Th2 cytokines. For example, we calculated the mean of IL-5 adjusting for IL-13 and found that the adjusted IL-5 (by IL-13) was not significantly associated with atopy. Similarly, when we fitted IL-5 and IL-13 together in a logistic regression model using atopy as the dependent variable, we did not find a significant association of these cytokines with atopy, possibly due to collinearity. These results (data not shown) were expected considering the pathogenic relationship of Th2 cytokines and further indicated that the adjustment of IL-10 and IFN-*γ* for Th2 cytokines is reliable and meaningful. 

Our community population was recruited in Perth, Australia, a developed country with a high prevalence of allergic diseases [13]. In our population, we found that Th2 cytokines distorted the association of IL-10 and IFN-*γ*with allergic phenotypes. However, in a Th1-dominant milieu, Th1 cytokines may also distort the association of Th2 cytokines with complex diseases of interest. This aspect requires further confirmation. 

IL-10 is a pleiotropic cytokine with regulatory effects on Th1/Th2 balance [4, 5, 19]. The precise functional role of IL-10, however, has not been clarified. Whether IL-10 expression is altered in asthmatics is uncertain, as it has been reported to be reduced in some studies but increased in others [11, 20, 21]. In a high-risk cohort, we have previously reported that the ratio of IL-10 and IL-5 was protective against the susceptibility to respiratory infections during infancy and early childhood [19]. In the current study, we found that adjusted IL-10 expression was clearly decreased in children with atopy or high total serum IgE levels. However, in individuals with asthma and wheezing, the effects of IL-10 still remain unclear, indicating the complex effects of regulatory cytokines in relation to the pathogenesis of asthma. 

In conclusion, in a western-community-based cohort, levels of Th2 cytokines distorted the association of IL-10 and IFN-*γ* with allergic phenotypes. After adjustment for Th2 cytokines, both IL-10 and IFN-*γ* were protective against atopy. 

## Figures and Tables

**Table 1:** Correlation coefficients between cytokines stimulated by HDM.

	IL-10	IFN-*γ*	IL-5	IL-13	IL-9	IL-4
IL-10	1					
IFN-*γ*	0.615**	1				
IL-5	0.321**	0.268**	1			
IL-13	0.453**	0.543**	0.746**	1		
IL-9	0.340**	0.360**	0.694**	0.762**	1	
IL-4	0.141	0.124	0.505**	0.479**	0.593**	1

**P < 0.01.

**Table 2:** Correlation coefficients between cytokines stimulated by PHA or SEB.

	PHA				SEB			
	IL-10	IFN-*γ*	IL-5	IL-13	IL-10	IFN-*γ*	IL-5	IL-13
IL-10	1				1			
IFN-*γ*	0.726**	1			0.553**	1		
IL-5	0.558**	0.567**	1		0.400**	0.102	1	
IL-13	0.407**	0.415**	0.652**	1	0.424**	0.130	0.623**	1

**P < 0.01.

**Table 3:** Raw and adjusted IL-10 and IFN-r levels (log value) stimulated with HDM in children with and without atopy.

	Atopy (*n* = 71)			Nonatopy (*n* = 59)			*P*
	Mean	95% CI		Mean	95% CI		
		Lower	Upper		Lower	Upper	
IL-10 (HDM)							
No adjustment	2.33	2.10	2.56	2.62	2.33	2.91	0.11
By IL-5	2.22	2.02	2.42	2.75	2.47	3.03	**0.002**
By IL-13	2.28	2.09	2.47	2.68	2.42	2.95	**0.012**
By IL-9	2.27	2.06	2.48	2.70	2.41	2.99	**0.016**
By IL-4	2.29	2.07	2.52	2.67	2.37	2.97	**0.043**

IFN-r (HDM)							
No adjustment	2.48	2.15	2.80	2.93	2.45	3.40	0.113
By IL-5	2.34	2.04	2.64	3.09	2.64	3.54	**0.005**
By IL-13	2.38	2.11	2.66	3.04	2.66	3.42	**0.005**
By IL-9	2.43	2.12	2.74	3.00	2.53	3.47	**0.037**
By IL-4	2.46	2.12	2.80	2.96	2.46	3.45	0.091

**Table 4:** Raw and adjusted IL-10 and IFN-r levels (log value) stimulated with either PHA or SEB in children with and without atopy.

	Atopy (*n* = 71)			Nonatopy (*n* = 59)			*P*
	Mean	95% CI		Mean	95% CI		
		Lower	Upper		Lower	Upper	
IL-10 (PHA)							
	*n* = 71			*n* = 58			
No adjustment	4.67	4.45	4.88	4.99	4.79	5.19	**0.030**
By IL-5	4.63	4.47	4.80	5.03	4.86	5.20	**0.001**
By IL-13	4.64	4.45	4.82	5.03	4.83	5.22	**0.004**
IL-10 (SEB)	*n* = 69			*n* = 58			
No adjustment	5.47	5.31	5.62	5.70	5.51	5.89	0.062
By IL-5	5.43	5.29	5.57	5.75	5.58	5.92	**0.004**
By IL-13	5.45	5.32	5.59	5.72	5.54	5.89	**0.017**

IFN-r (PHA)							
	*n* = 70			*n* = 59			
No adjustment	6.11	5.84	6.38	6.50	6.29	6.71	**0.026**
By IL-5	6.07	5.87	6.28	6.55	6.37	6.74	**0.001**
By IL-13	6.08	5.85	6.31	6.54	6.34	6.75	**0.003**
IFN-r (SEB)	*n* = 69			*n* = 58			
No adjustment	10.01	9.84	10.17	10.09	9.91	10.27	0.50
By IL-5	10.00	9.83	10.16	10.10	9.92	10.28	0.39
By IL-13	10.00	9.83	10.17	10.10	9.92	10.27	0.44

**Table 5:** Correlation coefficients between raw or adjusted IL-10 and IFN-*γ* and total IgE.

	Stimulated with					
	HDM		PHA		SEB	
	Coeff.	*P*	Coeff.	*P*	Coeff.	*P*
IL-10						
Raw	−0.114	0.20	−0.070	0.43	−0.084	0.35
By IL-5	−0.244	**0.005**	−0.260	**0.003**	−0.233	**0.009**
By IL-13	−0.214	**0.015**	−0.17	**0.055**	−0.190	**0.033**
By IL-9	−0.184	**0.045**				
By IL-4	−0.142	0.12				
IFN-*γ*						
Raw	−0.143	0.11	−0.085	0.34	−0.091	0.31
By IL-5	−0.249	**0.004**	−0.285	**0.001**	−0.125	0.16
By IL-13	−0.279	**0.001**	−0.190	**0.032**	−0.119	0.18
By IL-9	−0.189	**0.038**				
By IL-4	−0.140	0.13				

**Table 6:** Odds ratios of IL-10 and IFN-*γ* for atopy estimated by two adjusted methods*.

	Method one				Method two			
	OR	95% CI		*P*	OR	95% CI		*P*
		Lower	Upper			Lower	Upper	
IL-10 stimulated with HDM (HDM)								
Raw	.762	.544	1.066	0.11				
By IL-5	.481	.307	.755	**0.001**	.567	.390	.825	**0.003**
By IL-13	.602	.402	.902	**0.014**	.614	.415	.909	**0.015**
By IL-9	.628	.427	.924	**0.018**	.631	.430	.926	**0.019**
By IL-4	.693	.484	.992	**0.045**	.693	.485	.992	**0.045**
IL-10 (PHA)								
Raw	.615	.392	.963	**0.034**				
By IL-5	.401	.226	.712	**0.002**	.403	.227	.715	**0.002**
By IL-13	.472	.278	.802	**0.005**	.489	.293	.817	**0.006**
IL-10 (SEB)								
Raw	.607	.357	1.031	0.065				
By IL-5	.416	.224	.770	**0.005**	.427	.232	.784	**0.006**
By IL-13	.480	.259	.890	**0.020**	.489	.267	.894	**0.020**
IFN-*γ* (HDM)								
Raw	.838	.673	1.043	0.114				
By IL-5	.635	.473	.853	**0.003**	.720	.567	.914	**0.007**
By IL-13	.670	.503	.893	**0.006**	.681	.515	.900	**0.007**
By IL-9	.769	.599	.987	**0.039**	.772	.603	.988	**0.040**
By IL-4	.822	.655	1.033	0.092	.822	.655	1.033	0.093
IFN-*γ* (PHA)								
Raw	.645	.433	.963	**0.032**				
By IL-5	.437	.260	.734	**0.002**	.433	.256	.732	**0.002**
By IL-13	.502	.311	.812	**0.005**	.517	.323	.827	**0.006**
IFN-*γ* (SEB)								
Raw	.836	.500	1.399	0.50				
By IL-5	.788	.467	1.330	0.37	.794	.472	1.337	0.39
By IL-13	.815	.485	1.368	0.44	.815	.484	1.370	0.44

*Method one: “Raw” model only includes one independent variable: either raw IL-10 or IFN-*γ*; “by IL-5” includes either raw IL-10 or IFN-*γ* and IL-5; “by IL-13” includes either raw IL-10 or IFN-*γ* and IL-13; “by IL-9” includes either raw IL-10 or IFN-*γ* and IL-9; “by IL-4” includes either raw IL-10 or IFN-*γ* and IL-4. Method two: either adjusted IL-10 or IFN-*γ* was fitted in the logistic regression model.; “by IL-5”, “by IL-13”, “by IL-9”, and “by IL-4”indicate that the levels of either adjusted IL-10 or IFN-*γ* was adjusted by IL-5, IL-13, IL-9, and IL-4, respectively.
